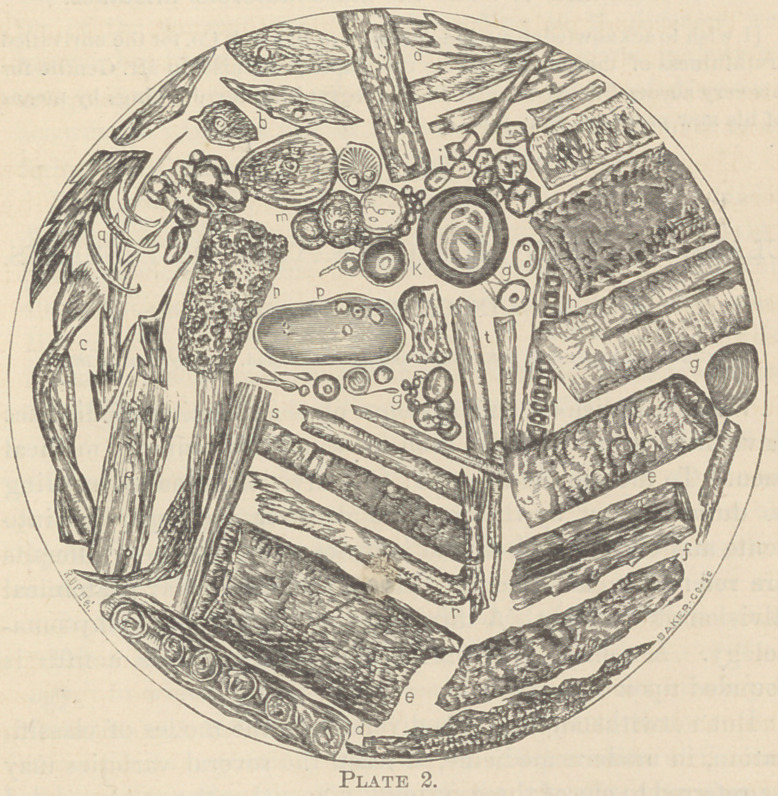# On the Pictorial Art in Illustrating Microscopic Observations, with Original Drawings and Descriptions

**Published:** 1877-03

**Authors:** R. U. Piper

**Affiliations:** Chicago


### EXAMINER.

Vol. XXXIV.—MARCH, 1877.—No. 3.

### Original Communications.

### ON THE PICTORIAL ART IN ILLUSTRATING MICROSCOPIC OBSERVATIONS, WITH ORIGINAL DRAWINGS AND DESCRIPTIONS.

By R. U. PIPER, M.D., Chicago.

A knowledge of the arrangement of material forms in their varied relations can only be conveyed from the observer to others through the actual exhibition of objects, or by means of accurate drawings. Language, if it possessed power to this end, is subject to such wide differences of interpretation that even to experienced observers it often fails to impart correct knowledge. As the material exhibition to any extent of the results of our studies is impossible, we must resort to the pictorial art if we desire that our discoveries should reach the world of science. Art, used to convey ideas, becomes the most potent means of instruction, not only telling what the pen cannot tell, but so condensing knowledge as to convey in a single figure what it would take whole pages to describe, even where such description is possible to language.

Dr. Beale remarks that "an honest inquirer cannot be of greater use in his time than by making good drawings of what he has seen, and that we may feel sure that those who follow us will respect our drawings, if honest copies of nature."

Any advance in our knowledge of a large number of diseases must rest mainly upon histological research; and here, of course, is required the use of the microscope.

Dr. Richardson says, "that at least one-half the cases of disease which the physician is called upon to treat would have some light thrown upon their nature by a careful examination of the renal secretion, sputum, blood, etc., with the microscope." It seems obvious, then, that an earnest and conscientious practitioner of medicine can scarcely discharge his whole duty to himself and his patients without frequent resort to such investigations.

Take for illustration kidney diseases, which are so common and so frequently grave. They may in many cases be treated with entire success if taken in their early stages. Owing to the labors of Bright, Johnson, Beale and others, the proper treatment of many of these diseases is now well understood. Most, if not all, of the advance in the knowledge of the treatment of these much dreaded disorders, has been made through the agency of the microscope. Through the liberality of the publishers of the JOURNAL AND EXAMINER I am able to show, by means of plates, what can now be done in this way to put on record discoveries made with the microscope, and by other methods also. I speak advisedly when I say *can now be done*, for heretofore, in too many cases where attempts have been made in this way to show minute structure, such attempts have resulted in failure.

In the present case the wood engraver has proved himself able to give "*wash drawings*" with almost absolute fidelity; as nearly so, I think, as may be done by the difficult and expensive process of aqua-tint etching.

So important is the need of giving texture in histological drawings that Dr. Beale devotes pages in urging its necessity, and in endeavoring to give directions for its accomplishment.

Such *fac simile* engravings, when the original drawing is correct, cannot fail to aid the student in his researches with the microscope.

I have chosen for the illustration of my subject two plates drawn by myself directly from the microscope; one of them illustrating a case of chronic Bright's disease, under the care of Dr. I. N. Danforth; the other showing foreign matter found in urine. It may be well, perhaps, in order to show the reliability of the drawings, to give in brief the method of their production. The usual way has been to outline the object by means of the camera-lucida, and afterwards to draw the shading and texture from the memory, a fancy of the artist. Dr. Beale says we cannot expect the artist to spend time drawing things which he neither knows nor desires, perhaps, to know anything about, and yet he tells us it is quite impossible to obtain a good representation of a microscopic object without long and careful study, and that it is necessary to give texture as well as outline in many cases, in order to the recognition of the object even. In such statements I think we may find the reason why so many errors in the texture and otherwise may be observed in the drawings in his magnificent volumes. There is another reason for a want of truth in texture and detail which may be observed in a large proportion of the published plates of microscopic observations. They have been drawn from low powers; hence, of course, minute structures could not be given. My course has been, in all cases where practicable, to use high powers when making the drawings, and to have them reduced on the engraver's block by means of the photographic process; that is, they are photographed on the wood from the negative taken from the drawing, as in copying on paper or any other substance. This enables me to preserve in its integrity every feature of the original picture.

I do not make *pencilled* outlines of my drawings, except in a few cases where it is necessary in order to obtain absolutely accurate measurements in drawing blood corpuscles, with reference to legal cases; but the drawings are made and finished under the camera with the brush. This allows the giving of texture, which is here so beautifully and for the first time, to my knowledge, faithfully copied by any wood engraver. The most exact method of making copies of drawings is by the photographic process, and it is best to resort to this method when but few copies are needed, as in reporting the results of a microscopic examination for individual study. But the use of the photographic process to produce images direct from the microscope for the purpose of histological investigation I think must in most cases prove an entire failure.

In studying morbid changes in so small an object as a blood corpuscle even, I am obliged to keep my finger nearly all the time on the fine adjustment screw of the microscope, and I do not remember a single drawing made with any degree of finish in which I have not had to change the focus a number of times during the progress of the work; nor, indeed, have I seen a single photograph taken in this manner in which the *whole* truth was depicted; *i. e.*, what could be easily observed by changing the focus of the instrument during the progress of the study.

Take the plate illustrating renal disease, as showing certain other difficulties in the way of rendering micro-photography of value.

As many as twenty dippings from the same specimen of urine were used in making up the plate; and if it were possible to bring all the objects seen in it together in one field, the difficulty would remain of photographing the different objects, many of them occupying different focal planes. There are other obstacles in the way of this process, such as the color of objects, etc., and which need not be detailed.

Regarding the use of high powers, Dr. Beale says, in the last edition of his book, "that they are absolutely necessary to the study of the minute structure of organized matter." I have myself long been convinced of this fact.

In studying blood, all my observations have been made with a power giving 1,275 diameters; and in observations on renal diseases, cancers, etc., it is necessary, in most cases, at some stages of the investigation, to resort to the use of such high powers.

Often, with the exclusive use of low powers, objects would be wrongly named or entirely overlooked, which would at once attract attention when highly magnified.

In both of the accompanying plates may be seen objects which could not have been recognized, or scarcely seen even, by a power of 200 diameters. Let any one try to examine blood discs under this power, and they will at once perceive how inadequate it is for the purpose. Recently a slide of dried

PLATE 1.

In Plate 1, *a* indicates air bubbles, two of them resting upon an epithelial cell; *b*, oil globules; *c c*, epithelial cells; *d*, vibriones; *e*, torula cerevisiæ; *g*, granular casts from the tubuli uriniferi; *h*, a mass of deep orange colored crystals, perhaps chloride of ammonium. Fragments of casts, etc., are seen scattered over various parts of the plate.

human blood was submitted, with the remark that it had been examined under such a power, and found to contain a large proportion of white blood discs. Upon examination with a high power, barely the normal number could be discovered. To-day, we have under examination a specimen of urine which contains bodies the most practiced observer would try in vain to distinguish under a power of 350 diameters; with a power of 1,200 they are seen to belong to certain species of diatomes. These bodies are frequently found in urine. They are doubtless introduced through the medium of the water used in rinsing the vials which contain the specimens. The bodies referred to might easily be mistaken for a cast undergoing disintegration.

The second plate contains objects often met with in the urine, which are introduced by accident or design. All observers agree as to the great importance of being able to recognize such extraneous substances.

They should be studied by themselves under different powers, being put into urine for the purpose, and then submitted to examination in the usual manner upon the glass slide.

Those shown in the plate have most of them been found in samples of urine under examination. All of them have been drawn while in urine, and thus they are made to appear as they would under the ordinary conditions in which they come under the notice of the medical practitioner. They were magnified much larger than they appear in the plate, and are reduced, as before described, from the original drawings.

Anyone who will take the trouble to compare these figures with those found in the books will perceive quite a difference in the form and texture of most of them. Portions of a feather are represented by *a a; b* indicates a group of five epithelial cells; *c*, three pieces of cotton fibre, a piece of hemp being seen at the right of the group; *d*, two pieces of pine wood; *e e*, human hair, the upper piece showing disease; *f*, portion of the root and shaft of dog's hair; *g g g*, groups of potato starch. This starch is seen under these three forms: with the waved surface, with the nucleus, and as in the central group; *h* marks four pieces of cat's hair, all cut from a single specimen. The smallest piece of this hair is given as the typical form in Beale's plate; corn starch is shown at *i;* air bubbles at *k*, with other forms beneath and attached at the side; *m* is a group of wheat starch, one of the forms presenting a very curious appearance; *n*, portion of a rice grain, with a mass of cooked wheat flour lying to the left of it. This piece of rice grain might well be mistaken for a cast. It is best in working to have a watery solution of iodine to test such forms when there is any doubt in the case. A group of oil globules is seen at *p*, some of them resembling spermatozoa. These globules are described in the books as being yellow. This depends upon the kind of oil composing them. In this case, and in most

PLATE 2.

cases I meet with, they present a sort of pearly hue. Four pieces of linen fibre are shown at *r;* *s* is a piece of wool fibre; *t*, silk fibres. Several plates might be made, showing foreign bodies which are frequently found in urine.

We have found clay, brick dust, ocherous earth, etc., etc., in samples of urine, often introduced by accident, sometimes, doubtless, for purposes of deception. Recently I met with diatoms from the Pacific coast. These came from sea-weed which had been sent in a letter. Pieces of tea leaves, tobacco, and such substances are quite common. Epithelial cells and other substances from the mouth, introduced through the medium of the sputum may cause the observer some trouble. The want of a careful study of such foreign matters under the conditions in which they are found, has "caused the observer sometimes to make the most ludicrous mistakes."

[I wish to acknowledge my indebtedness to Baker & Co. for the unrivalled truthfulness of the engravings in this paper; and also to Mr. Gentilé for his very successful experiment in photographing my drawings by means of his new process.—R. U. P.]

## Figures and Tables

**Plate 1. f1:**
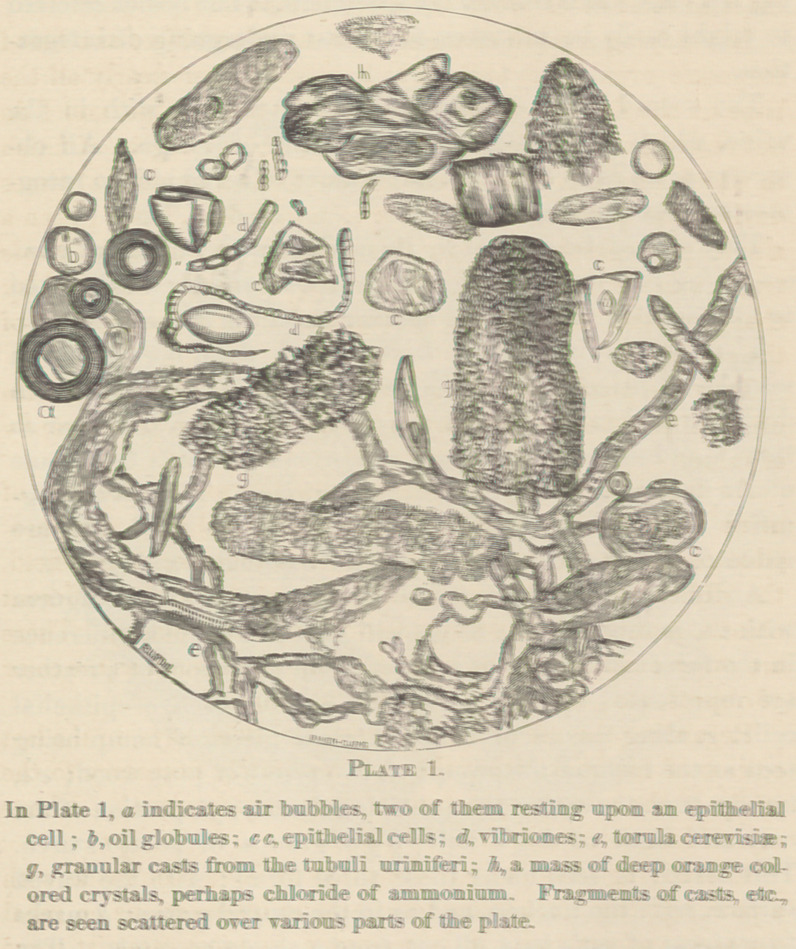


**Plate 2. f2:**